# Semantic-based Privacy-preserving Record Linkage.

**DOI:** 10.23889/ijpds.v7i3.1956

**Published:** 2022-08-25

**Authors:** Yang Lu

**Affiliations:** 1 York St John University

## Introduction

Sharing aggregated electronic health records (EHRs) for integrated health care and public health studies is increasingly demanded. Patient privacy demands that anonymisation procedures are in place for data sharing.

## Objective

Traditional methods such as k-anonymity and its derivations are often overgeneralising resulting in lower data accuracy. To tackle this issue, we proposed the Semantic Linkage K-Anonymity (SLKA) approach to balance the privacy and utility preservation through detecting risky combinations hidden in the record linkage releases.

## Approach

K-anonymity processing quasi-identifiers of data may lead to ‘over generalisation’ when dealing with linkage data sets. As most linkage cases do not include all local patients and thus not all modifying data for privacy-preserving purposes needs to be used, we proposed the linkage k-anonymity (LKA) by which only obfuscated individuals in a released linkage set are required to be indistinguishable from at least k-1 other individuals in the local dataset. Considering the inference disclosure issue, we further designed the semantic-based linkage k-anonymity (SLKA) method through extending with a semantic-rule base for automatic detection of (and ruling out) risky associations from previous linked data releases. Specially, associations identified from the “previous releases” of the linkage dataset can become the input of semantic reasoning for the “next release”.

## Results

The approach is evaluated based on a linkage scenario where researchers apply to link data from an Australia-wide national type-1 diabetes platform with survey results from 25,000+ Victorians about their health and wellbeing. In comparing the information loss of three methods, we find that extra cost can be incurred in SLKA for dealing with risky individuals, e.g., 13.7% vs 5.9% (LKA, k=4) however it performs much better than k-anonymity, which can cause 24% information loss (k=4). Besides, the k values can affect the level of distortion in SLKA, such as 11.5% (k=2) vs 12.9% (k=3).

## Conclusion

The SLKA framework provides dynamic protection for repeated linkage releases while preserving data utility by avoiding unnecessary generalisation as typified by k-anonymity.

